# B Cell Orchestration of Anti-tumor Immune Responses: A Matter of Cell Localization and Communication

**DOI:** 10.3389/fcell.2021.678127

**Published:** 2021-06-07

**Authors:** Gabriela Sarti Kinker, Glauco Akelinghton Freire Vitiello, Wallax Augusto Silva Ferreira, Alexandre Silva Chaves, Vladmir Cláudio Cordeiro de Lima, Tiago da Silva Medina

**Affiliations:** ^1^Translational Immuno-oncology Group, International Research Center, A.C. Camargo Cancer Center, São Paulo, Brazil; ^2^Department of Pathological Sciences, Londrina State University, Londrina, Brazil; ^3^Laboratory of Tissue Culture and Cytogenetics, Environment Section (SAMAM), Evandro Chagas Institute, Ananindeua, Brazil; ^4^Instituto do Câncer do Estado de São Paulo (ICESP), São Paulo, Brazil; ^5^Oncologia D’Or São Paulo, Rede D’or, São Paulo, Brazil; ^6^National Institute of Science and Technology in Oncogenomics and Therapeutic Innovation, São Paulo, Brazil

**Keywords:** B lymphocytes, tertiary lymphoid structures, germinal center, T lymphocytes, tumor, anti-tumor responses, pro-tumor responses

## Abstract

The immune system plays a crucial role in cancer development either by fostering tumor growth or destroying tumor cells, which has open new avenues for cancer immunotherapy. It was only over the last decade that the role of B cells in controlling anti-tumor immune responses in the tumor milieu has begun to be appreciated. B and plasma cells can exert anti-tumor effects through antibody-dependent cell cytotoxicity (ADCC) and activation of the complement cascade, even though their effector functions extend beyond the classical humoral immunity. In tumor tissues, B cells can be found in lymphoid aggregates, known as tertiary lymphoid structures (TLSs), well-organized non-encapsulated structures composed of immune and stromal cells. These structures reflect a process of lymphoid neogenesis occurring in peripheral tissues upon long-lasting exposure to inflammatory signals. The TLS provides an area of intense B cell antigen presentation that can lead to optimal T cell activation and effector functions, as well as the generation of effector B cells, which can be further differentiated in either antibody-secreting plasma cells or memory B cells. Of clinical interest, the crosstalk between B cells and antigen-experienced and exhausted CD8^+^ T cells within mature TLS was recently associated with improved response to immune checkpoint blockade (ICB) in melanoma, sarcoma and lung cancer. Otherwise, B cells sparsely distributed in the tumor microenvironment or organized in immature TLSs were found to exert immune-regulatory functions, inhibiting anti-tumor immunity through the secretion of anti-inflammatory cytokines. Such phenotype might arise when B cells interact with malignant cells rather than T and dendritic cells. Differences in the spatial distribution likely underlie discrepancies between the role of B cells inferred from human samples or mouse models. Many fast-growing orthotopic tumors develop a malignant cell-rich bulk with reduced stroma and are devoid of TLSs, which highlights the importance of carefully selecting pre-clinical models. In summary, strategies that promote TLS formation in close proximity to tumor cells are likely to favor immunotherapy responses. Here, the cellular and molecular programs coordinating B cell development, activation and organization within TLSs will be reviewed, focusing on their translational relevance to cancer immunotherapy.

## Introduction

Cancer arises from the accumulation of genetic mutations that ultimately lead to global epigenetic changes within transformed cells. These genetic and epigenetic alterations can potentially give rise to neoantigens whose recognition by the immune system is fundamental for tumor control. In particular, CD8^+^ T cells exert potent anti-tumor activities through the recognition of tumor neoantigens presented on class I major histocompatibility complex (MHC-I) molecules. However, when governed by an immunosuppressive microenvironment, immune cells may seed a fertile niche for tumor growth (Jones et al., 2019). Among the established hallmarks of cancer, tumor-promoting inflammation and tumor evasion from the immune response have emerged as important processes for cancer development and progression (Hanahan and Weinberg, 2011). The recognition that the immune system can counterintuitively be involved in either cancer development or control, depending on the milieu, represented a milestone in cancer research and has led to significant breakthroughs in clinical care for a growing list of malignancies (Farkona et al., 2016).

The field of immuno-oncology, however, is not as new as one might think: evidence demonstrating that the immune system is capable of eliminating tumors dates back to the early civilizations, when historical reports have documented spontaneous regression of tumors associated with concomitant infections and fever. In the nineteenth century, Rudolf Virchow was the first pathologist to observe the presence of inflammatory cells in solid tumors. Later, William Coley’s pioneering empirical work reported tumor shrinkage in cancer patients who had severe postoperative infections. This groundbreaking observation led him to inoculate *Streptococcus pyogenes* into tumors to promote inflammation and tumor regression. Reaching the 1950s and 1960s, transplantation of chemically induced tumors between syngeneic animals pointed to the existence of anti-tumor-specific immunity endowed with memory (Dobosz and Dzieciatkowski, 2019).

Since then, leveraged by the outstanding technical and scientific progress made in molecular biology and immunology, the mechanisms behind tumor recognition by the immune system have been dissected (Dobosz and Dzieciatkowski, 2019). Over the past years, cytotoxic CD8^+^ T lymphocytes have been in the center of this debate, as their anti-tumor response can optimally eliminate tumor cells by specifically recognizing tumor antigens that originate from mutated or aberrantly expressed proteins linked to MHC-I on the surface of tumor cells (Schumacher and Schreiber, 2015).

Further advances in immunology have helped to elucidate why CD8^+^ T cells often fail to efficiently control the development of cancer, revealing that immune regulatory mechanisms operate in the cancer milieu. The inflammatory counterbalance promoted by immune regulatory mechanisms is essential to prevent undesirable local tissue damage. Accordingly, multiple immune regulatory processes can be induced in the tumor microenvironment and include, but are not limited to, suboptimal priming of lymphocytes by immature dendritic cells maintained in the absence of inflammatory signals, tolerance mechanisms triggered by autoantigens expressed in tumor cells, as well as T cell exhaustion due to chronic antigenic stimulation (van der Leun et al., 2020). This accumulated knowledge paved the way for the development of several therapies aimed to overcome these barriers, including cytokine therapies, cellular vaccines employing *ex-vivo* activated dendritic cells, and immune checkpoint blockade (ICB) therapies, which are currently approved for several types of cancers (Zhang and Zhang, 2020).

Despite the major impact that immunotherapies, specially ICB, have had on cancer treatment, several challenges still persist for their widespread application, as evidenced by the significant number of patients that do not derive clinical benefits and the cancer types wherein their efficacy is still to be proved. Thus, further clarification of the immune mechanisms operating in the tumor microenvironment is needed in order to identify novel therapeutic targets and biomarkers of response (Gibney et al., 2016; Bai et al., 2020).

As previously highlighted, cytotoxic T lymphocytes, thought to be the major effector cells in anti-tumor immune responses, have received the greatest attention throughout the history of the immuno-oncology field. However, more recently it has become apparent that these cells are not capable of acting independently, relying on a complex network of interactions with other immune and stromal cells for their coordinated activation, maintenance and function (Garner and de Visser, 2020). In this context, the pivotal role of B lymphocytes on anti-tumor immune responses has only begun to be appreciated. These cells can mediate both pro-and anti-tumor effects and perform a plethora of roles in the tumor milieu, such as secretion of antibodies and cytokines, antigen presentation for both cytotoxic (CD8^+^) and helper (CD4^+^) T lymphocytes, and coordination and maintenance of lymphoid aggregates known as tertiary lymphoid structures (TLS), which are privileged sites for antigen presentation and T cell (re-)activation (Sautes-Fridman et al., 2019; Sharonov et al., 2020).

In this review, we outline the multiple functions of B lymphocytes in anti-tumor immune responses. In particular, we briefly describe B cell biology from genesis to effector functions. We also discuss the spatial organization of B cells in the tumor microenvironment, highlighting the molecular mechanisms that promote B and T cell compartmentalization within TLSs in chronic inflammatory processes, focusing mainly on cancer. We then present evidence for both pro-and anti-tumor responses of B cells depending on what cellular niche these cells occupy. We also describe how the spatial organization within TLS can shape anti-tumor effector B and T cell responses and we provide evidence that mature TLSs are an ideal environment capable of triggering optimal T cell activation, which ultimately leads to the expression of clinically relevant immune checkpoints.

## B Lymphocyte Biology: From B Cell Ontogeny to Activation and Its Role in Antigen Presentation

B lymphocytes are the main cellular components of the humoral compartment of adaptive immunity (Sebina and Pepper, 2018). Their development occurs mainly in the liver during fetal life and in the bone marrow (BM) after birth through several sequential steps of differentiation, wherein hematopoietic stem cells (HSCs) reach the common lymphoid progenitor (CLP) stage, which gives rise to either B, T or Natural Killer (NK) cells (Rieger and Schroeder, 2012). B lymphocytes derived from the fetal liver belong to the B-1a lineage and comprise a population of long-lived and self-renewing B lymphocytes that occupy body cavities and mucosae and constitutively secret IgM antibodies (“natural antibodies”) of restricted specificity (Baumgarth, 2017). The vast majority of B lymphocytes in adults is derived from the BM and can be divided into the B-1b lineage, which are functionally similar to B-1a cells, and the B-2 lineage, which migrate from the BM to the spleen to further differentiate into marginal-zone or follicular B lymphocytes (Pieper et al., 2013).

The ontogeny of lymphocytes initiates with the differentiation of HSCs into a lymphoid-primed multipotent progenitor (LMPP), which expresses the lymphoid-specific recombinases RAG1 and RAG2 that promote LMPP differentiation into the earliest lymphoid progenitor (ELP) and then into CLPs. In mice, the commitment of the B cell lineage is critically dependent on the expression of the cytokine receptors FLT3 and IL-7R (Medina and Singh, 2005), while IL-7 signaling does not seem to be essential in humans (Pieper et al., 2013).

The early transcription factors (TFs) involved in B lymphocyte commitment, E2A and EBF, act coordinately to repress key molecular programs from other cell lineages and promote the expression of PAX5, which further stabilizes lineage commitment, thus defining the pro-B stage (Sigvardsson, 2018). Next, developing B cells begin to mount their antigen specific B-cell receptors (BCR) that are constituted by accessory signaling proteins coupled to transmembrane isoforms of IgM antibodies. These are composed of two heavy chains generated through recombination between variable regions (V_H_, D_H_, and J_H_) from the Ig heavy (H) locus, each combined with a light chain, generated through recombination between variable regions VL and JL from Ig κ or λ light (L) chain *loci* (Jung and Alt, 2004). This process generates approximately 5×10^13^ B lymphocyte clones, each expressing a unique BCR that recognizes a different epitope (Pieper et al., 2013).

Initially, E2A and EBF1 direct RAG1 and RAG2 recombinases to the IgH locus in pro-B cells, promoting D_H_-J_H_ recombination (Romanow et al., 2000). Then, V_H_-DJ_H_ recombination is facilitated by PAX5-promoted “contraction” of IgH *locus* (Fuxa et al., 2004; Jung et al., 2006). The terminal deoxynucleotidyl transferase (TdT) is specifically expressed during this phase and further increases the junctional diversity by adding random nucleotides in recombination sites (Bertocci et al., 2006). After recombination, IgH forms a complex with the polypeptide chains V Pre-B and λ5, which substitute the Ig light chain. IgH also interacts with Igα (CD79a), and Igβ (CD79b), giving rise to the pre-BCR, whose expression is regulated by the key TFs that characterize the pre-B stage (Sigvardsson, 2018).

The correct assembly of pre-BCR represents an important checkpoint for B cell development. This receptor signals through the Burton’s tyrosine kinase (BTK) and the adapter protein BLNK to promote cell survival, proliferation and activation of the TFs NF-κB and interferon response factor 4 (IRF4), which have been shown to be essential for the rearrangement of IgL chains. The recombination events are initiated in Ig κ locus and followed by λ locus if κ rearrangements for both alleles result in a non-functional or self-reactive BCR (Pieper et al., 2013; Sigvardsson, 2018). Complex molecular mechanisms ensure that Ig rearrangements for IgH and IgL occur at one allele at a time, promoting the usage of the other allele only if the first recombination is unproductive. The mechanisms behind this process, named allelic exclusion, are not yet fully understood (Vettermann and Schlissel, 2010).

Developing B cells that successfully produce non-self-reactive BCRs undergo molecular changes including a decrease in the chemokine receptor CXCR4 signaling (Beck et al., 2014) and an increase in the expression of sphingosine-1-phosphate receptor 1 (S1P1) (Cinamon et al., 2004), which mediate the migration of B cells to the spleen, where they complete their maturation to marginal zone or follicular B cells, depending on the strength of BCR signaling to self-antigens and NF-κB, BAFF, and Notch2 signaling pathways (Pillai and Cariappa, 2009). Marginal-zone B cells remain on the spleen mediating mainly T-independent responses to blood-borne antigens from diverse chemical natures (proteins, lipids, carbohydrates); whereas follicular B cells migrate through B-cell zones (or follicles) in lymph nodes throughout the body, guided by the interaction between CXCR5, expressed on their cell surface, and CXCL13, a chemokine produced by follicular dendritic cells (FDCs). Then, the encounter between naïve B lymphocytes and their cognate antigens, either captured from peripheral tissues by FDCs or freely borne through lymph, is favored in follicular B-cell zones (Cyster and Allen, 2019).

As part of the innate immune response, complement system-derived peptides can bind to the surface of pathogens/antigens. FDCs capture complement-covered pathogens and antigens in peripheral tissues through complement-specific receptors present on their cell membranes and transport them to lymph nodes. In follicles, BCR interacts with surface antigens, and high-affinity interactions promote both BCR signaling and internalization of the bound antigen through a clathrin-dependent mechanism. Activation of co-receptors on B cell surface, such as complement receptors and pathogen-associated molecular pattern recognition receptors, such as Toll-like receptors (TLRs), amplifies BCR signaling and diminishes the B cell activation threshold, while inhibitory receptors that recognize self-molecules, such as CD22, or antibodies, such as FcγRIIb, may increase the threshold for B cell activation, acting as a negative feedback to prevent auto-reactivity and exacerbated immune responses (Cyster and Allen, 2019).

As a consequence of early B cell activation, these cells downregulate the expression of S1P1 and begin to express CCR7 and EBI2, which direct them to the interface between B cell follicles and adjacent T cell zones, following CCL21 and CCL19 gradients. In parallel, these cells process the internalized antigen to present antigen-derived peptides bound to MHC-II on their cell surface along with co-stimulatory ligands, such as CD80/CD86 and ICOSL. Altogether, these phenotypic changes favor B cell interaction with activated CD4^+^ helper T cells migrating in the opposite direction. The recognition of MHC-II-bound peptides by CD4^+^ T cells along with co-stimulatory signals promote their activation and differentiation into follicular-helper T cells (T_FH_), which express CD40L, that interact with CD40 on B cells. This intimate B-T cell interaction in the presence of cytokines, such as IL-4 and IL-21, promotes B cell survival, proliferation and activation (Cyster and Allen, 2019).

Activated B cells also undergo class-switch recombination (CSR), in which the conserved region of expressed antibodies is changed from those derived from Cμ and Cσ loci to those coded by Cγ, Cα, or Cε loci. This allows the replacement of IgM and IgD by IgG, IgA, or IgE, depending on the inflammatory stimulus. Each conserved region holds different effector functions and is induced by different cytokines secreted by T_FH_ cells. For instance, IFN-γ promotes IgG2a and IgG3 production, while IL-4 facilitates switching to IgE, and TGF-β favors IgA (Zhang, 2003). CSR is depends on the activated induced deaminase (AID) expressed in B cells, which also promotes hypermutation of the Ig variable region, further increasing antibody diversification and affinity maturation in a clonal fashion (Cyster and Allen, 2019).

In summary, B cells capture, process and present antigens to CD4^+^ T cells, what may lead to mutual activation. Once

activated, B cells undergo proliferation while changing their antibody repertoire by inducing class-switching and affinity maturation. This drives the development of a germinal center (GC), composed of a “dark zone” (DZ), where B cells interact with T_FH_ cells and undergo activation and clonal expansion, and a “light zone” (LZ), characterized by cells derived from the DZ undergoing CSR and affinity maturation. In the LZ, cells that effectively produce high-affinity antibodies differentiate either into antibody-secreting plasma cells or memory B cells (Gatto and Brink, 2010).

**FIGURE 1:**
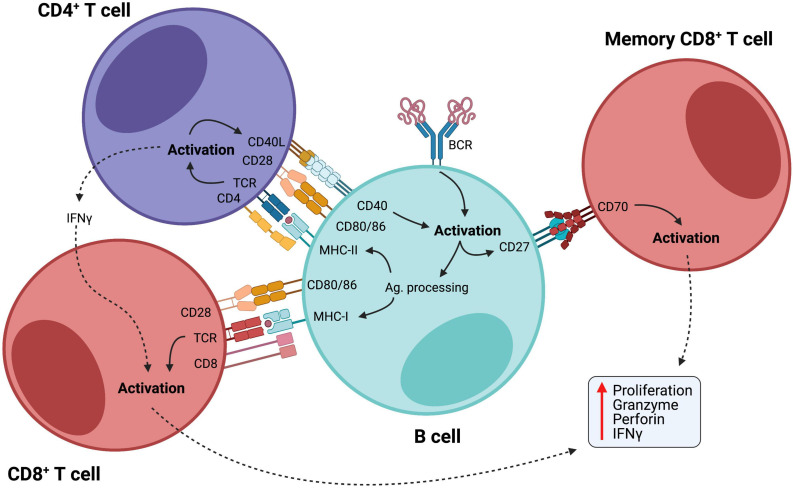
B cells as activators of cellular immunity. B cells are initially activated by antigen recognition through BCR. Internalized antigens are then presented through class II MHC to CD4^+^ helper T cells, which provide co-stimulatory signals for B cell activation. Activated B cells acquire enhanced potential for antigen presentation with upregulation of MHC-I and II and co-stimulatory molecules, such as CD80/86, further activating both CD4^+^ and CD8^+^ T cells. Also, CD27 is upregulated in activated B cells, and interaction between this molecule and CD70 on the membrane of memory CD8^+^ T cells promotes their maintenance and facilitates their activation in an antigen-independent manner. CD8^+^ T cell activation leads to efficient cell proliferation and production of potent inflammatory mediators, such as granzymes, perforin, and IFN-γ.

The ability of B cells to efficiently present antigens and activate T cells through the expression of co-stimulatory molecules is of particular interest, as a large body of evidence has shown that B cells can present antigens as efficiently as professional APCs, such as dendritic cells, and promote T cell mediated immune responses in various contexts (Rivera et al., 2001; Chen and Jensen, 2008; de Wit et al., 2010; Adler et al., 2017; Hong et al., 2018), including cancer (Nielsen et al., 2012; Rossetti et al., 2018), where B cells demonstrated high capacity of cross-presenting internalized antigens via MHC-I to activate cytotoxic T cells (Gnjatic et al., 2003). Additionally, the interaction between CD27-expressing B cells and CD70-expressing CD8^+^ T lymphocytes induces cytotoxic T cell responses in an antigen-independent manner (Deola et al., 2008), demonstrating another way by which B cells stimulate T cell responses (Figure 1).

## Secondary Lymphoid Organs and Tertiary Lymphoid Structures: Functionally Similar, But Slightly Different in Composition

The formation of TLSs is a phenomenon associated with chronic inflammatory conditions, such as autoimmune diseases and cancer, reflecting a process of lymphatic neogenesis that creates a local hub for antigen presentation and immune responses (Pitzalis et al., 2014). Though the main cellular components of TLSs are lymphocytes and dendritic cells, the adequate organization of this complex environment is sustained by a network of non-hematopoietic stromal cells. Populations of lymphatic and blood endothelial cells and immunofibroblasts are at the core of this framework (Buckley et al., 2015).

Key steps involved in the formation of secondary lymphoid organs (SLOs) during organogenesis are also commonly observed in the formation of TLSs. However, while SLO development occurs in predefined areas during embryogenesis, TLSs are ectopically formed at sites of chronic inflammation. A crucial step for the fetal development of the secondary lymphoid tissue is the migration of integrin α4β7^+^ lymphoid tissue inducer cells (LTis) that interact with MadCAM-1^+^ high endothelial venules (HEVs) in the lymph node anlagen (Mebius et al., 1996). LTis originate from common lymphoid progenitors in the fetal liver and undergo maturation through a process involving the Notch signaling pathway and the transcriptional repressor Id2. This generates terminally differentiated LTis expressing RORγT and α4β7 integrin (Cherrier et al., 2012). Retinoic acid produced by nerve endings allows mesenchymal cells to express the chemokine CXCL13, whose gradient attracts LTis via interaction with the receptor CXCR5. Although both CXCL13 and CCL21 chemokines have the capacity of attracting LTis during the early embryonic phases, only CXCL13 is expressed in all lymph node anlagen at that time (van de Pavert et al., 2009).

Once properly recruited to the anlagen, LTis activate stromal lymphoid tissue organizer cells (LTos) through the interaction between the lymphotoxin (LT) α1β2 and its receptor, LTβR. This favors the expression of several adhesion molecules by LTos, such as VCAM-1, ICAM-1, and MadCAM-1, which, together with the homeostatic chemokines CCL19, CCL21, and CXCL13, promotes the recruitment of lymphocytes and the retention of LTis (Peduto et al., 2009; Vondenhoff et al., 2009). Another interesting point is that while chemokines promote the recruitment of DCs, NK, T and B cells, LTβR signaling aids the development of lymph nodes, as well as cell clustering and spatial organization. LTos develop further into follicular (FC) and fibroblastic reticular cells (FRC) that provide the conduit framework on which T and B cells migrate and interact with each other, allowing the proper development of adaptive immunity (Veiga-Fernandes et al., 2007; van de Pavert et al., 2009).

Much of the current knowledge about TLS formation and function comes from murine models of autoimmunity (Pipi et al., 2018). In human Sjogren’s Syndrome (SS) and murine models of SS, TLS assembly is strictly dependent on a network of gp38^+^ immunofibroblasts that are phenotypically and functionally similar to FRC networks in SLO. In both cases, the coordinated action of IL-22, LTα1β2, and Th2 cytokines seems to be associated with such network formation. Although lymphocytes are not needed for the priming of immunofibroblast progenitors, they contribute for the network expansion and activation. While RORγT^+^ LTis play a critical role in the development of fibroblast reticular cells in SLOs, they are not required for priming and expansion of TLS immunofibroblasts in SS (Nayar et al., 2019). Of note, IL-21-producing Th17 cells might work as LTi cells that initiate TLS organogenesis and GC formation (Deteix et al., 2010). A murine model of experimental autoimmune encephalomyelitis (EAE) demonstrated that gp38^+^ Th17 cells stably producing Th17 cytokines induce the expression of CXCL13 in the central nervous system and the development of follicle-like structures (Peters et al., 2011). These structures were composed of B cell clusters, surrounded by a mantle of T cells and collagen fibers; the latter could extend into the center of B cell clusters. Moreover, the expression of GC markers GL7 and PNA revealed the existence B cell aggregates with different levels of maturation.

### Germinal Center Maturation in Chronically Inflamed Scenarios

**FIGURE 2:**
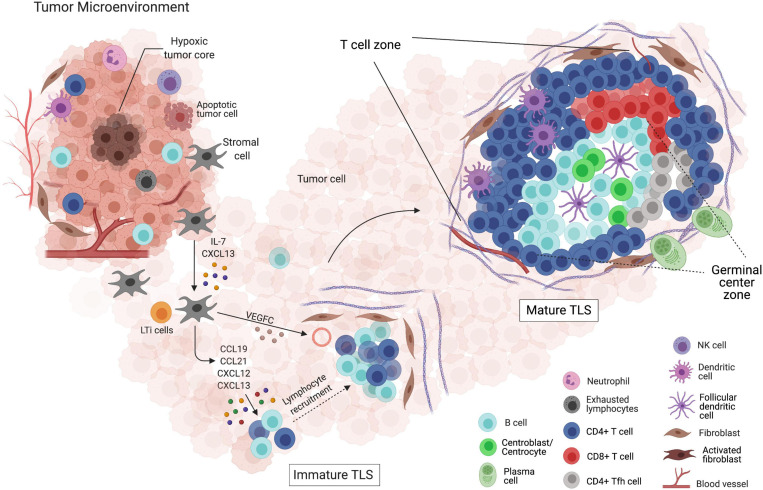
Formation and maturation of tertiary lymphoid structures (TLSs) within the tumor microenvironment. (I) Depiction of the tumor microenvironment and its interactions with both innate and adaptive immune cells, and the non-immune components. (II) LTis attracted by the CXCL13 gradient, produced by activated stromal cells, migrate to the site of TLS initiation. Chemokines, such as CCL19, CCL21, CXCL12, and CXCL13, allow the migration and retention of additional LTis along with lymphocytes. The gathering of leukocytes surrounded by an extracellular matrix net produced by fibroblasts and newly formed blood vessels supports immature TLS formation, which fails to mount effective anti-tumor responses. (III) The presence of clearly delimited T cell and B cell zones, as well as a GC-like structure, defines mature TLSs. In this structure, the induction of plasma cells and memory B cells takes place, reflecting the interaction with other specific cell types, such as FDCs, T_FH_, and CD8^+^ T cells. Surrounded by pericytes and expressing adhesion molecules, the normal high endothelial venules (HEVs) located at the TLS vicinity favor the formation of mature TLSs, which are thought to be a rich niche of anti-tumor B and T cell responses.

So far, we have presented a sketch of the TLS structure, briefly pointing out what makes them different from SLOs. However, we have not delved into a particular aspect, common to both structures, that may draw special attention: the GC maturation (Figure 2). Classical markers of GC B cells include lack of surface IgD, upregulation of CD38, high expression levels of Fas and n-glycolylneuraminic acid, along with the selective expression of BCL-6 and AID. When mature, GCs constitute well-structured regions with two dynamic compartments: the DZ and LZ. The gradient of CXCL12 and CXCL13, along with the expression of their respective receptors CXCR4 and CXCR5, dictates the spatial distribution of B cells. During the events taking place into GCs, activated B cells acquire new features and functions. The CXCR4-expressing centroblasts are mostly located in the DZ, due to the CXCL12 abundance, whereas the CXCR5-expressing centrocytes move to the LZ, in favor of the CXCL13 gradient (Gatto and Brink, 2010). Besides moving through the GC zones in response to a chemokine gradient, B cells can also be engaged in short, dynamic interactions with both T cells and antigens.

This whole process can be observed in well-structured TLS, as they are analogous in structure and function to SLOs (Germain et al., 2014). In immature TLSs, on the other hand, B cells cannot interact and expand properly, hampering the formation of active GCs. Indeed, in early steps of hepatocellular carcinoma progression, immature TLSs are associated with inefficient immune responses and tumor evasion (Meylan et al., 2020). Maturation of murine TLSs can be induced by LT-producing B cells, as it has been shown that the reconstitution of LTα^–/–^ recipients with bone marrows containing LT-producing B cells was critical for skewing immature TLSs into mature TLSs (Lorenz et al., 2003; McDonald et al., 2005).

In colorectal cancer, multi-parameter immunofluorescence detection of CD21, CD23 and CXCL13 revealed three different states of TLS maturation: (i) early TLS as dense lymphocytic aggregates; (ii) primary follicle-like TLS, composed of B cell clusters with FDC networks, but devoid of GCs; and (iii) the secondary follicle-like TLS, including active GCs with CD23^+^ B cells (Posch et al., 2018). In melanoma samples, mature GCs composed of highly proliferative KI67^+^ B cells were associated with higher frequencies of antigen-experienced CD4^+^ T cells expressing the anti-apoptotic molecule BCL2 (Cabrita et al., 2020). Such structures also included TCF7^+^IL7R^+^ naive and/or memory CD4^+^ and CD8^+^ T cells. In a cohort of esophago-gastric primary adenocarcinomas, approximately 48% of patients presented TLSs along the tumor invasive margin, as well as tumor-specific antibodies in the serum, underscoring the presence of B cell-dependent anti-tumor responses (Schlosser et al., 2019). In line with these observations, CD3^+^CD4^+^CCR7^–^CD45RA^–^CXCR5^+^ T_FH_ cells and CD20^–^CD27^+^CD38^+^ plasmablasts were found to be enriched in tumor samples compared to peripheral blood. Interestingly, the number of B cells was reduced in the microenvironment of tumors that expressed PD-L1 or lacked the expression of HLA-I (Schlosser et al., 2019).

In autoimmune diseases, spontaneous GC development often contributes to the disease severity due to self-reactive B cells (Domeier et al., 2017). In this case, IFN-γ plays a major role through its signaling pathway, as IFNγR signaling phosphorylates STAT1 and upregulates T-bet in B cells, which is required for spontaneous GC formation and class switching to IgG2b and IgG2c antibodies (Domeier et al., 2016). A different perspective can be seen in the context of chronic infectious diseases. In a C57BL/6 mouse model of *Mycobacterium tuberculosis* infection with H37Rv or HN878 strains (Hertz et al., 2020), sex affected the appropriate formation of B cell follicles upon infection, as chronically H37Rv-infected male mice expressed diminished levels of CXCL13 and CCL19 in the lungs, in comparison with female mice. Besides, when HN878 strain infection developed, there was a significantly higher amount of IL-17A, IL-23, and IL-1β in female lungs (Hertz et al., 2020). Perhaps in this scenario, sex differences between hosts and their genetic background may impact the assembly of mature TLSs (Hertz and Schneider, 2019). In a *Helicobacter pylori* infection model, CXCR5^–/–^ mice failed to induce the formation of TLSs and Peyer’s patches, which generated less antigen-loaded DCs, diminished T cell priming and impaired T cell-dependent B cell response against the pathogen (Winter et al., 2010).

But what should be expected when cancer-related scenarios are considered? We will next explore in detail the biological and clinical relevance of the spatial organization of B cells in different types of tumors. We will also speculate how this system may be exploited clinically to improve patient prognosis and responses to immunotherapy.

## Pro-Tumor Functions of B Lymphocytes

**FIGURE 3:**
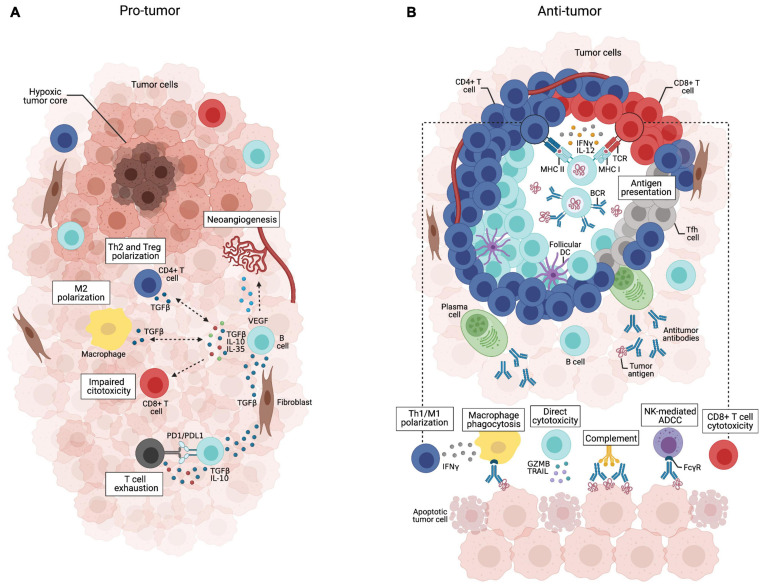
Dual role of tumor-infiltrating B cells. **(A)** B cells scatteredly distributed throughout the tumor bulk may acquire immunosuppressive phenotypes in response to stimulation with TGF-β secreted by fibroblasts, Tregs or M2 macrophages. They can release IL-10, IL-35, and TGF-β that support Treg expansion and Th2/M2 polarization, while suppressing effector T cell activity, which is potentialized by B cell PD-L1 expression. VEGF-producing B cells may also promote tumor progression through neoangiogenesis.**(B)** Tumor-infiltrating B cells organized in well-structured TLSs coordinate anti-tumor immune responses through multiple mechanisms. They can present tumor-derived antigens to T cells and secrete cytokines such as IFN-γ and IL-12 that support Th1/M1 polarization and CD8^+^ T cell cytotoxicity. Tumor-specific antibodies secreted by plasma cells can trigger the complement cascade, mediate phagocytosis of tumor cells, and antibody-dependent cell cytotoxicity by NK cells. Activated B cells may also directly kill tumor cells by secreting TRAIL and granzyme B.

Our understanding of the multifaceted functions of B lymphocytes in tumor immunity has improved substantially in recent years. Current evidence suggests that the tumor microenvironment may have distinct B cell subpopulations that can exert both pro– or anti–tumor activities (Germain et al., 2014; Shimabukuro-Vornhagen et al., 2014; Shalapour et al., 2015; Wouters and Nelson, 2018; Figure 3), hence affecting patient outcomes (Mauri and Menon, 2017; Hu et al., 2019). The balance between the dual role of B cells is influenced by several factors, such as hypoxia (Caro-Maldonado et al., 2014; Shin et al., 2014; Meng et al., 2018), cytokines and metabolites produced by tumor cells (Wejksza et al., 2013; Pimenta et al., 2015; Ricciardi et al., 2015; Somasundaram et al., 2017; Ye et al., 2018), other immune cells [e.g., regulatory T cells (Tregs) and myeloid-derived suppressor cells (MDSC); Zhao et al., 2006; Shen et al., 2018; Wang Y. et al., 2018], inhibitory factors produced by B cells (Kessel et al., 2012; Khan et al., 2015; Shalapour et al., 2015) and immune checkpoints (Nishimura et al., 1998; Okazaki et al., 2001; Haas, 2011; Escors et al., 2018).

When outside the TLS, B cells can acquire a plethora of suppressive functions, meaning that B cell spatial distribution across the tumor microenvironment influences their activities. Thus, it is tempting to speculate that B cells acquire pro-inflammatory features that fully activate T cell responses when compartmentalized within TLS, while B cells outside the TLS are likely to acquire anti-inflammatory features that contribute to tumor growth (Cabrita et al., 2020). Within TLSs, it is possible that the mantle of T cells physically protects B cells from immunosuppressive stimuli found in the tumor tissue. The recent advent of technical approaches capable of interrogating the spatial architecture of tumor tissues (e.g., spatial transcriptomics) will allow the detailed exploitation of the TLS composition and function (Vickovic et al., 2019). Therefore, identifying strategies that reconfigure the spatial architecture of immune cells in the tumor microenvironment might have a great impact on the outcome of cancer patients.

Several studies have provided solid evidence that the tumor-promoting effects are mainly led by a diverse population of B cells known as regulatory B cells (Breg) (Schwartz et al., 2016; Sarvaria et al., 2017; Matsushita, 2019; Sharonov et al., 2020; Wang et al., 2020). Multiple Breg phenotypes that fulfill an immunosuppressive role have been described in different human solid tumors (Lee-Chang et al., 2013; Shao et al., 2014; Zhou et al., 2014; Wang et al., 2015; Wei et al., 2016; Roya et al., 2020; Wu et al., 2020a), as well as in peritumoral tissues (Garaud et al., 2019), peripheral blood (Qian et al., 2015; Wang K. et al., 2018; Karim and Wang, 2019; Murakami et al., 2019) and tumor-draining lymph nodes (Ganti et al., 2015), suggesting their broad clinical relevance and potential therapeutic application. Nevertheless, due to intricate origins and activation pathways, there is still no clear consensus on Breg-cell-specific phenotypic or lineage commitment markers, and the transcription factors that specifically drive the development of these cells remain elusive (Oleinika et al., 2019; Wang et al., 2020).

In both human and mice, most studies of Bregs are concentrated in memory CD27^+^ and transitional CD38^+^ B cells, sharing markers such as IgA^+^CD138^+^ and IgM^+^CD147^+^ with plasma cells (Fremd et al., 2013; Schwartz et al., 2016). Recently, a solution to this problem was proposed employing Toll-like receptor ligands, together with high dimensional data-analysis (Chaye et al., 2021). The available data suggest that Breg cells can arise at various stages during B cell development and differentiation in response to various cues, including cytokines (e.g., IL-35, IL-21, IL-1β, and IL-6) (Yoshizaki et al., 2012; Rosser et al., 2014; Wang et al., 2014; Dambuza et al., 2017), large amounts of calcium influx (Matsumoto et al., 2011; Matsumoto and Baba, 2013) and activation of surface molecules such as TLRs, CD40 and BCRs (Fillatreau et al., 2002; Yoshizaki et al., 2012; Rosser et al., 2014; Menon et al., 2016; Ran et al., 2020).

Breg cells support carcinogenesis, tumor progression and metastasis predominantly, although not exclusively, through the production of IL-10, TGF-β, and IL-35 or by intercellular contact (Ammirante et al., 2010; Shao et al., 2014; Tao et al., 2015; Wang et al., 2015; Schwartz et al., 2016; Xiao et al., 2016; Sarvaria et al., 2017). IL-10-producing Bregs can induce dendritic cells to produce IL-4 and downregulate IL-12, thereby affecting the Th1/Th2 balance (Moulin et al., 2000); they can suppress CD4^+^ T cell differentiation into Th1 (suppressing IFN-γ and TNF-α production) and Th17 (suppressing IL-17 production); they also suppress TNF-α-producing monocytes (Iwata et al., 2011). Bregs can also induce the polarization of naïve CD4^+^T cells into both FOXP3^+^ Treg cells and IL-10-producing Tr1 cells, modulating cancer progression and increasing tumor metastasis (Olkhanud et al., 2011). Additionally, Bregs can promote apoptosis of effector CD4^+^ T cells through the expression of FasL (Fillatreau et al., 2002; Iwata et al., 2011; Matsumoto et al., 2014; Wang et al., 2015; Zhou et al., 2016). Furthermore, IL-10-producing Breg cells have been shown to contribute to tumor progression by positively regulating the differentiation of tumor-associated macrophages (TAMs), skewed toward a M2 macrophage phenotype, that ultimately inhibit effector T and NK cells (Rosser et al., 2014; Wu et al., 2020b). It is currently unclear whether Bregs actively promote tumor growth, or an increase in the Breg population merely reflects the immune response against the tumor (Sarvaria et al., 2017).

Breg cells have also been reported to have other immunosuppressive mechanisms, such as (i) B cell expression of programmed cell death-1 (PD-1) via TLR4 activation, which induces T cell dysfunction and foster cancer progression; (ii) expression of other inhibitory molecules such as programmed death-ligand 1 (PD-L1) and FasL, which regulate humoral immunity mediated by CD4^+^CXCR5^+^PD-1^+^ T_FH_ cells via PD-1; (iii) combined secretion of IgG4 and IL-10; (iv) IL-21–mediated induction of GZMB, which efficiently suppresses T cell proliferation by GrB-dependent TCR-ζ degradation; (v) production of adenosine 5’-monophosphate (AMP) and adenosine (ADO), which suppresses activated T cells; (vi) activation of geranylgeranyl pyrophosphate (GGPP) (an intermediate of cholesterol metabolism), permitting transduction of signaling cascades necessary for IL-10 expression; (vii) production of indoleamine 2,3-dioxygenase (IDO), which inhibits T cell responses; (viii) expression of Aryl hydrocarbon receptor (AhR), which modulates the differentiation and function of Bregs, specially by suppressing their pro-inflammatory transcriptional program; and (ix) TLR4-mediated BCL6 upregulation that induces T cell dysfunction through PD-1 expression and IL-10 secretion (Gotot et al., 2012; Lindner et al., 2013; Saze et al., 2013; van de Veen et al., 2013; Khan et al., 2015; Nouel et al., 2015; Ren et al., 2016; Xiao et al., 2016; Piper et al., 2019; Bibby et al., 2020). Furthermore, glioma cell-derived placental growth factors (PIGFs) have been shown to promote proliferation of intratumoral B cells by inducing their differentiation into TGF-β-producing Bregs that suppress CD8 T cell anti-tumor activities (Han et al., 2014).

Akin to murine studies, other human B cells are key mediators of tumor growth. In melanoma, gastric, lung, liver and prostate cancers, tumor-infiltrating B cells can persistently express VEGF (vascular endothelial growth factor) and other pro-angiogenic genes via STAT3 signaling, fostering tumor progression by increasing angiogenesis (Yang et al., 2013). Another subset of STAT3-activated B cells found in human prostate, non-small cell lung, and ovarian cancers, CD5^+^ B cells promote tumor progression upon IL-6 activation (Zhang et al., 2016). Furthermore, IL35-producing B cells (CD1d^*high*^CD5^+^) are required to support growth of early pancreatic neoplasia, more specifically KRAS^*G1*2D^-harboring neoplastic lesions (Pylayeva-Gupta et al., 2016). In addition, a hypoxic tumor microenvironment restrains the TLS formation and limits tumor elimination. Indeed, pancreas-specific hypoxia-inducible factor 1α (HIF1α) deletion increased CXCL13 secretion and B-cell infiltration and was associated with accelerated tumor growth (Lee K.E. et al., 2016). This supports the idea that a compromised tumor microenvironment is likely to disrupt the spatial architecture of immune cells, largely perturbing the migration of immune cell populations and their organization in privileged sites, particularly TLSs, that elicit optimal activation and maintenance of anti-tumor immune responses. There is now great interest in understanding whether intratumoral perturbations, including aberrant neoangiogenesis, hypoxia, acidification, intense cell death by necrosis or apoptosis and fibrosis, could negatively impact TLS formation. Furthermore, CD19^+^ B cells in metastatic ovarian carcinoma and CD20^+^ and CD138^+^ B cells infiltration in epithelial ovarian cancer were associated with poor outcome (Dong et al., 2006; Lundgren et al., 2016). Taken together, a better understanding of immunosuppressive B cell subpopulations and their underlying roles may open new avenues for cancer immunotherapy.

## Anti-tumor Role of TLS B Cells

Accumulating evidence indicates that TLSs play a major role in controlling tumor progression (Teillaud and Dieu-Nosjean, 2017; Figure 3). Overall, despite the heterogeneity of methods used for quantifying TLSs, studies have consistently found that high densities of intra and peritumoral TLSs are associated with prolonged overall survival and disease-free survival in more than 10 types of malignancies (Table 1), including sarcoma (Petitprez et al., 2020), melanoma (Cabrita et al., 2020), lung (Dieu-Nosjean et al., 2008; Germain et al., 2014; Tang et al., 2020; Rakaee et al., 2021), breast (Lee H.J. et al., 2016; Liu X. et al., 2017), colorectal (Coppola et al., 2011; Vayrynen et al., 2014) and pancreatic cancers (Hiraoka et al., 2015; Castino et al., 2016). The cellular composition and spatial organization of tumor-associated TLSs indicate that the development of B cell-dependent anti-tumor immunity lay the basis for the contribution of such structures to a favorable prognosis.

**TABLE 1:** B cell and TLS presence and abundance as prognostic factors in different tumors.

Author, year	Tumor type	N	Sample type	Method of assessment	Finding

B lymphocytes					
Ladanyi et al. (2011)	Cutaneous melanoma	106	FFPE	Immunohistochemistry	High number of CD20^+^ B cells (intratumoral and peritumoral) associated with improved OS
Mahmoud et al. (2012)	Breast cancer	1,470	FFPE	Immunohistochemistry	Higher total CD20^+^ B cell counts associated with better DFI and BCSS
Woo et al. (2014)	Prostate carcinoma	53	FFPE	Immunohistochemistry	Intratumoral CD20^+^ B cells associated with cancer recurrence and progression
Germain et al. (2014)	Lung cancer	74 early stage 122 advanced stage	FFPE	Immunohistochemistry	High density of follicular CD20^+^ B cells within TLSs associated with better OS
Castino et al. (2016)	Pancreatic cancer	104	FFPE	Immunohistochemistry	High density of B cells within TLSs associated with improved DSS.
Miligy et al. (2017)	Breast DCIS	36	FFPE	Immunohistochemistry	High number of CD20^+^ B cells associated with shorter RFI
Sakimura et al. (2017)	Gastric cancer	226	FFPE	Immunohistochemistry	High number of CD20^+^ B cells associated with longer OS
Arias-Pulido et al. (2018)	Inflammatory breast cancer	221	FFPE	Immunohistochemistry	CD20^+^PD-L1^+^ lymphocytes were an independent favorable prognostic factor for DFS and BCSS
Edin et al. (2019)	Colorectal	316	FFPE	Multiplexed immunohistochemistry and multispectral imaging	High number of CD20^+^ B cells associated with improved DSS
Murakami et al. (2019)	Gastric cancer	59	FFPE	Double staining immunohistochemistry (CD19 and IL-10)	Regulatory B cells (CD19^+^IL10^+^) associated with worse 5-year OS rate
Chen et al. (2020)	NK/T-cell lymphoma	56	FFPE	Immunohistochemistry	High density of CD20^+^ B cells associated with improved OS
Petitprez et al. (2020)	Sarcoma	496	STS public datasets (TCGA SARC, GSE21050, GSE21122 and GSE30929)	Gene expression (TME deconvolution)	B cell signature associated with improved OS

**TLS**					

Coppola et al. (2011)	Colorectal cancer	21	Fresh tumor	Microarray	Higher expression of a 12-chemokine TLS signature in long-term survivors
Vayrynen et al. (2014)	Colorectal cancer	418 (cohort 1)	FFPE	H&E	Higher TLS density (the number of follicles/the length of the invasive front) associated with improved 5-year survival
Hiraoka et al. (2015)	Pancreatic cancer	308	FFPE	Immunohistochemistry	Higher relative area of intratumoral TLSs associated with improved OS and DFS
Schweiger et al. (2016)	Colorectal cancer (lung metastases)	57	FFPE	Immunohistochemistry	The presence of TLSs was not associated with improved RFS or OS
Lee H.J. et al. (2016)	Resected triple negative breast cancer	769	FFPE	H&E	Moderate or abundant TLSs associated with better DFS
Liu X. et al., 2017	Invasive breast cancer	248	FFPE	Immunohistochemistry	Presence of TLS associated with improved DFS in HER2^+^ tumors
Silina et al. (2018)	Resected squamous cell lung carcinoma	138	FFPE	H&E	Number of TLSs per mm^2^ was the strongest prognostic factor
Calderaro et al. (2019)	Resected hepatocellular carcinoma	273	FFPE	H&E	Presence of intratumoral TLSs associated with lower risk of early tumor relapse following surgery
Sofopoulos et al. (2019)	Ductal breast carcinoma	112	FFPE	H&E	Patients with peritumoral TLSs had worse DFS and OS
Cabrita et al. (2020)	Cutaneous melanoma	117	FFPE	Immunohistochemistry	Presence of TLSs and tumor associated CD8^+^ cells associated with improved OS
Li et al. (2020)	Resected oral cancer	65	FFPE	H&E	Patients whose tumors were enriched for intratumoral TLSs had better DFS and OS
Tang et al. (2020)	Lung cancer	133	FFPE	Immunohistochemistry	High TLS number per mm^2^ and relative area associated with improved 10-year survival
Rakaee et al. (2021)	Lung cancer	553	FFPE	Immunohistochemistry	TLS score was an independent positive prognostic factor of DFS and OS, regardless of the quantification strategy used (four-scale semi-quantitative; absolute count of total TLSs; absolute count of total TLSs with germinal center)

*TLS, tertiary lymphoid structures; FFPE, formalin-fixed paraffin-embedded samples; DFI, disease-free interval; BCSS, breast cancer specific survival; RFI, relapse-free interval; DCIS, ductal carcinoma in situ; DSS, disease-specific survival; OS, overall survival; STS, soft tissue sarcoma; RFS, recurrence-free survival; H&E, hematoxylin and eosin staining.*

The presence of active GCs with AID^+^ and BCL-6^+^ B cells, as well as differentiated memory B cells and plasma cells, has been detected within TLSs from diverse cancer types (Cipponi et al., 2012; Germain et al., 2014; Kroeger et al., 2016; Montfort et al., 2017; Garaud et al., 2019). Analysis of the repertoire of immunoglobulins in melanoma (Cipponi et al., 2012; Helmink et al., 2020), ovarian cancer (Kroeger et al., 2016) and invasive breast ductal carcinoma (Nzula et al., 2003) samples demonstrated B cell clonal amplification, somatic hypermutation (SHM) and CSR, revealing a local antigen-driven response and antibody affinity maturation. Similarly, a comprehensive characterization of immune cells from triple-negative breast cancer patients using paired single-cell RNA and TCR/BCR sequencing revealed that tumor-infiltrating B cells were mostly CD27^+^ memory B cells and had higher clonality, CSR and SHM than those in the blood (Hu et al., 2021). Unsupervised clustering further identified a group of AICDA^+^ and MKI67^+^ proliferative B cells and CD38^+^ plasma cells in tumor samples, underscoring the existence of functionally active GCs. Widespread B cell clonal expansions and immunoglobulin subclass switch events were also evidenced in multiple human cancers by a large-scale report, analyzing more than 30 million IgH complementarity-determining region 3 sequences assembled from ∼9,000 tumor RNA-seq samples (32 cancer types) in The Cancer Genome Atlas (TCGA, Hu et al., 2019).

Activated TLS B cells may modulate T cell phenotypes within the tumor microenvironment through their ability to present tumor-derived peptides. Multiple studies have shown that tumor-infiltrating B cells with an activated/memory phenotype express markers of antigen presentation, including MHC class I and II and costimulatory molecules such as CD40, CD80, and CD86 (Nielsen et al., 2012; Shi et al., 2013; Bruno et al., 2017; Rossetti et al., 2018). Although dendritic cells are the major APCs that provide initial T cell activation in the lymph nodes, antigen-presenting B cells can contribute to additional CD4^+^ T cell expansion intratumorally, as demonstrated by *ex vivo* co-culture assays (Bruno et al., 2017; Rossetti et al., 2018). Accordingly, in lung tumors, a high density of TLS B cells was associated with increased CD4^+^ T cell receptor repertoire clonality (Zhu et al., 2015) and with a reduced percentage of Tregs (Germain et al., 2021). The capacity of B cells to cross-present antigens to CD8^+^ T cells is also well established (Heit et al., 2004; Hon et al., 2005; Marino et al., 2012) and has been demonstrated in the context of the cancer testis antigen NY-ESO-1 (Gnjatic et al., 2003). Moreover, in ovarian cancer, antigen-experienced CD20^+^ B cells colocalized with activated CD8^+^ T cells, and the presence of both populations correlated with increased patient survival compared with the presence of CD8^+^ T cells alone (Nielsen et al., 2012). Memory B cells may also possess tumor-killing potential by producing IFN-γ, interleukin 12*p*40 (IL-12*p*40), granzyme B, and TRAIL (Shi et al., 2013).

Plasma cells generated within tumor-associated TLSs may reside locally and produce significant amounts of tumor-specific antibodies, as shown for breast cancer (Coronella et al., 2002; Pavoni et al., 2007), melanoma (Erdag et al., 2012), non-small cell lung cancer (Lohr et al., 2013; Germain et al., 2014), and high-grade serous ovarian cancer (Montfort et al., 2017). Importantly, the isotype and specificity of such antibodies can drive distinct immune responses (Sharonov et al., 2020). The IgG1 antibody class is of primary importance for anti-tumor cytotoxic responses, as these antibodies can bind to Fcγ receptors and trigger antibody-dependent cellular cytotoxicity and phagocytosis, complement activation, and enhance antigen presentation by dendritic cells (Gilbert et al., 2011; Boyerinas et al., 2015; Carmi et al., 2015). Accordingly, high intratumoral IgG1 has been associated with longer patient survival, while IgA may drive an opposite pattern (Welinder et al., 2016; Bolotin et al., 2017; Isaeva et al., 2019). Additionally, analysis of more than 5,000 TCGA RNA-seq samples revealed that high levels of IgG3–1 switch are associated with prolonged survival in patients with high SHM rates, whereas IgG3–1 levels are not prognostic in low SHM samples, underscoring the role of SHM in generating BCR sequences with high binding affinity to the exposed tumor antigens (Hu et al., 2019).

In prostate cancer and hepatocellular carcinoma, IgA-producing plasma cells have been shown to function as potent immunosuppressive cell populations through the expression of IL-10 and PD-L1 (Shalapour et al., 2015, 2017). On the other hand, in ovarian cancer, a recent report demonstrated that protective humoral responses are dominated by the production of polyclonal IgA, which binds to polymeric IgA receptors universally expressed on the tumor cells (Biswas et al., 2021). Interestingly, IgA responses were shown to impair ovarian cancer growth through complementary mechanisms: antigen-specific IgA redirected myeloid cells against cell-surface antigen-positive tumor cells, while transcytosis of non-antigen-specific IgA by tumor cells induced broad transcriptional changes, including the upregulation of IFN-γ receptors and several DUSP phosphatases, which antagonize the RAS pathway.

Accumulating evidence shows that the functional state of tumor-infiltrating B cells is shaped by whether they are organized or not in well-structured mature TLSs (Figure 3). Careful examination of tissue slides from adenocarcinoma of the pancreas (Castino et al., 2016) and the esophagogastric junction (Knief et al., 2016) revealed that a high density of CD20^+^ B cells was associated with increased patient survival, but only if B cells were organized within TLSs. Similarly, the abundance of CD20^+^ B cells in ovarian cancer was only associated with improved prognosis in the presence of the TLS marker CXCL13. CD20^*hi*^CXCL13^*hi*^ tumors had significantly longer overall survival and progression-free survival than the CD20^*lo*^ or CXCL13^*lo*^ counterparts (Yang et al., 2021). Regulatory phenotypes may arise when B cells are scattered throughout the stroma and have substantial interaction with malignant (Zhao et al., 2018) and TGFβ-producing cells, as suggested by the low T cell activity in immature TLSs (Cabrita et al., 2020). It is, thus, crucial to consider the spatial distribution of B cells to properly understand their dual role in controlling tumor progression. Importantly, experiments with pre-clinical murine models should be interpreted with caution, as fast-growing orthotopic tumors might develop a malignant cell-rich bulk with reduced stroma and devoid of TLSs (Spear et al., 2019).

It is noteworthy that TLS-rich tumors are typically more infiltrated by CD8^+^ T cells, which may express inhibitory immune checkpoints following persistent antigen exposure and/or inflammatory signals. In mature TLSs of sarcoma patients, the intimate contact between B and T cells was sufficient to induce high expression of the immune checkpoint PD-1 on T cells (Petitprez et al., 2020). Similarly, expression levels of the inhibitory receptors TIGIT and CTLA-4 were elevated in TLS^hi^ non-small cell lung cancer samples compared to the TLS^lo^ ones (Rakaee et al., 2021). Thus, TLSs may represent a rich site of expression of clinically relevant immune checkpoints.

## Clinical Applications and Future Perspectives

Recent studies have demonstrated a prominent association between B cell-dependent anti-tumor immunity and responsiveness to immunotherapy in different types of cancer. Analysis of post-treatment resection specimens of non-small-cell lung carcinoma patients in the first trial of neoadjuvant anti-PD-1 (nivolumab) showed that immune-mediated tumor clearance was characterized by local formation of TLSs and the presence of plasma cells (Cottrell et al., 2018). Notably, a TLS gene expression signature predicted clinical outcomes to ICB with anti-CTLA-4 and/or anti-PD-1 in multiple cohorts of melanoma samples (Cabrita et al., 2020). At baseline, melanoma-infiltrating B cells from responders were shown to have increased BCR clonality and diversity, as well as a higher proportion of CD27^+^ memory B cells (Helmink et al., 2020). A similar predictive role for B cells organized in TLSs was observed for ICB responses in patients with renal cell carcinoma (Helmink et al., 2020) and soft tissue sarcoma (Petitprez et al., 2020). Interestingly, higher expression of the TLS marker CXCL13 was independently associated with prolonged survival and objective response in muscle-invasive bladder cancer patients treated with ICB, while no significant results were observed for non–ICB-treated patients (Groeneveld et al., 2021).

Although mounting evidence highlights the central role of B lymphocytes and TLS in priming effective anti-tumor immune responses (Sautes-Fridman et al., 2019), they have largely been overlooked in clinical trials that have mostly focused on reversing T cell exhaustion. Except for drugs developed to treat B cell lymphomas and leukemias, clinical strategies that directly modulate B lymphocytes or harness the formation of TLSs have not yet been developed for solid tumors.

Barbera-Guillem et al. (2000) used rituximab to deplete B cells in a mouse model of liver metastasis of colorectal carcinoma (CRC) and demonstrated a reduction in the number of metastases following treatment. In the same study, fourteen metastatic CRC patients received rituximab, and among the 8 patients who completed treatment, half had tumor regression and 1 achieved stable disease as evaluated by positron emission tomography (Barbera-Guillem et al., 2000).

LIGHT (TNFSF14) is a cytokine important to the development and maintenance of SLOs and TLSs. In a preclinical murine model of pancreatic cancer, the administration of LIGHT linked to a vascular targeting peptide (LIGHT-VTP) promoted the formation of TLSs, stimulated intratumoral T cell infiltration, enhanced response to anti-PD1 and anti-CTLA4 therapies and strongly synergized with anti-tumor vaccination (Johansson-Percival et al., 2017).

In a phase I/II study (J0810) involving potentially resectable pancreatic cancer, 59 patients were randomized, with a 1:1:1 ratio, to receive GVAX, a GM-CSF-secreting irradiated pancreatic tumor vaccine, administered intradermally alone (arm A) or in combination with low dose intravenous (arm B) or oral cyclophosphamide (arm C) before and after surgery. Fifty-four patients underwent surgery, five of them did not have pancreatic adenocarcinoma, 1 had an ampullary cancer and 11 had cancer recurrence immediately following surgery, all these patients were excluded from further analysis. Among the remaining 39 patients, 33 (85%) developed intratumoral TLS, whereas none of 54 unvaccinated patients from a previous study had lymphoid aggregates in their tumors. Besides, vaccinated patients had improved antigen specific (anti-mesothelin) T cell response and enhanced T effector/T regulatory ratio. The authors did not include a control group treated with cyclophosphamide only, and despite they suggested that TLS formation was associated with longer survival; this result was underpowered and biased due to patient selection (Lutz et al., 2014). Recently, Zheng et al. (2021) updated the results of this trial after the inclusion of 38 additional patients (87 in total). Combining low dose cyclophosphamide with GVAX promoted worse disease-free survival (arm A × B × C: 18.92 × 8.54 × 5.56 months) and overall survival (34.2 × 15.4 × 16.5) compared to GVAX alone. Increased density of intratumoral TLS was associated with longer overall survival (Zheng et al., 2021).

In another study, metastatic pancreatic adenocarcinoma (PDAC) patients were treated in a random fashion (2:1 ratio) with two doses of GVAX followed by 4 doses of live-attenuated *Listeria monocytogenes* expressing mesothelin (CRS-207) (arm A) or 6 doses of GVAX plus cyclophosphamide (arm B). The combination of GVAX and CRS-207 improved median overall survival (primary endpoint) from 3.9 months in arm B to 6.1 months in arm A (HR 0.59; *p* = 0.02). Mesothelin-specific T cell number was higher in arm A as well. However, no objective responses were observed, and progression-free survival was similar in both arms, which makes it challenging to rule out an imbalance due to the effect of subsequent treatments in overall survival (Le et al., 2015).

On the other hand, Tsujikawa et al. (2020) randomized 93 metastatic PDAC patients to receive the same treatment mentioned above (GVAX+ low dose cyclophosphamide+ CRS− 207) with or without nivolumab (an anti-PD1 monoclonal antibody). They did not observe any difference in overall survival. The authors also analyzed 22 paired tumor samples before and after treatment and found an increase lymphoid cell density, an increased frequency of PD1-EOMES-CD8^+^ T cells and a decrease in CD68^+^ myeloid cells within the tumor microenvironment after cycle 3 in patients treated with GVAX and nivolumab (Tsujikawa et al., 2020).

Similarly, Wu A. A. et al. (2020) randomized 82 metastatic PDAC patients who obtained at least stable disease following 8–12 cycles of FOLFIRINOX (5-fluorouracil, leucovorin, irinotecan and oxaliplatin) to continue FOLFIRINOX or receive 4 doses of ipilimumab (an anti-CTLA4 monoclonal antibody) plus GVAX every 3 weeks. The study was stopped due to futility after an interim analysis; OS was shorter in the GVAX arm (9.38 × 14.7 months). Nevertheless, the authors observed an increase in effector and memory T cells in peripheral blood following treatment with GVAX+ ipilimumab. Similarly, they observed an increase in total CD4^+^ and CD8^+^ T cells, late effector memory CD8^+^ T cells and M1 macrophages and a decrease in CD4^+^FOXP3^+^ Tregs, early effector, exhausted CD8^+^ T cells and M2 macrophages in the tumor microenvironment comparing tumor samples obtained before and after treatment. However, it is difficult to tease out which intervention (GVAX alone, ipilimumab or the combination) promoted the aforementioned modifications.

On the other hand, corticosteroids, diet, rituximab and chemotherapy can impair the formation of TLS and reduce the development of GCs, potentially blunting anti-tumor immune response (Liu L. et al., 2017; Silina et al., 2018; Ryan et al., 2020).

Overall, these data indicate that strategies modulating TLS development in tumor milieu may hold promise in cancer treatment and extend the use of TLS and B cells beyond prognostic and therapeutic biomarkers to therapeutic targets. Studies in the basic research field aiming to further elucidate the mechanisms governing TLS evolution in cancer may pave the way for the development of new therapeutic approaches in this sense.

## Concluding Remarks

There is a growing appreciation that prominent anti-tumor immune responses are elicited when immune cells are spatially organized in privileged sites, namely TLSs, that facilitate cell-to-cell interaction and optimal antigen presentation. The detailed characterization of such complex intratumoral cellular networks is gaining momentum due to their clinical relevance, since TLSs can be used as a biomarker to classify cancer patients that are likely to benefit from immunotherapy approaches. More importantly, due to their capacity of mounting coordinated anti-tumor T and B cell responses, TLSs can be used to identify clinically relevant cancer-related immune checkpoints, and further research may reveal strategies capable of modulating TLS formation in the tumor microenvironment. Previously neglected, B cells are now considered key cellular components that initiate and sustain anti-tumor responses, as they appear to be indispensable T lymphocyte allies in the fight against cancer cells.

## Author Contributions

GK, GV, WF, and AC designed the figures. All authors wrote and approved the manuscript before submission.

## Conflict of Interest

The authors declare that the research was conducted in the absence of any commercial or financial relationships that could be construed as a potential conflict of interest.
